# Corrigendum: Mitochondrial ATP transporter depletion protects mice against liver steatosis and insulin resistance

**DOI:** 10.1038/ncomms16143

**Published:** 2017-08-18

**Authors:** Joonseok Cho, Yujian Zhang, Shi-Young Park, Anna-Maria Joseph, Chul Han, Hyo-Jin Park, Srilaxmi Kalavalapalli, Sung-Kook Chun, Drake Morgan, Jae-Sung Kim, Shinichi Someya, Clayton E. Mathews, Young Jae Lee, Stephanie E. Wohlgemuth, Nishanth E. Sunny, Hui-Young Lee, Cheol Soo Choi, Takayuki Shiratsuchi, S. Paul Oh, Naohiro Terada

Nature Communications 8 Article number: 14477 ; DOI: 10.1038/ncomms14477 (2017); Published: 02 16 2017; Updated: 08 18 2017

In the References section of this Article, incorrect papers are cited for references 4, 5, 7, 9, 10, 13–20 and 29–31. In addition, in the Methods section, reference 12 should be cited instead of reference 30 following the statement ‘Immunoblot analysis was performed as we described previously’, and reference 30 should be cited instead of reference 31 following the statement ‘while plasma glucose was maintained at basal concentrations as previously described’. The corrected references are listed below.

The list of corrections is as it follows.

· Ref #4 [Valianpour, F. *et al*. Monolysocardiolipins accumulate in Barth syndrome but do not lead to enhanced apoptosis. *J. Lipid Res.* **46**, 1182–1195 (2005)] is now [Monné, M. & Palmieri, F. Antiporters of the mitochondrial carrier family. *Curr. Top. Membr* **73**, 289–320 (2014)].

· Ref #5 [Gonzalez, I. L. Barth syndrome: TAZ gene mutations, mRNAs, and evolution. *Am. J. Med. Genet. A* **134**, 409–414 (2005)] is now [Rodić, N. *et al*. DNA methylation is required for silencing of ant4, an adenine nucleotide translocase selectively expressed in mouse embryonic stem cells and germ cells. *Stem Cells* **23**, 1314–1323 (2005)].

· Ref #7 [Winker, R. *et al*. Functional adrenergic receptor polymorphisms and idiopathic orthostatic intolerance. *Int. Arch. Occup. Environ. Health* **78**, 171–177 (2005)] is now [Esposito, L. A., Melov, S., Panov, A., Cottrell, B. A. & Wallace, D. C. Mitochondrial disease in mouse results in increased oxidative stress. *Proc. Natl Acad. Sci. USA* **96**, 4820–4825 (1999)].

· Ref #9 [Winker, R. *et al*. Endurance exercise training in orthostatic intolerance: a randomized, controlled trial. *Hypertension* **45**, 391–398 (2005)] is now [Brower, J. V., Lim, C. H., Jorgensen, M., Oh, S. P. & Terada, N. Adenine nucleotide translocase 4 deficiency leads to early meiotic arrest of murine male germ cells. *Reproduction* **138**, 463–470 (2009)].

· Ref #10 [Brinkman, J., de Nef, J. J., Barth, P. G. & Verschuur, A. C. Burkitt lymphoma in a child with Joubert syndrome. *Pediatr. Blood Cancer* **44**, 397–399 (2005)] is now [Lim, C. H., Brower, J. V., Resnick, J. L., Oh, S. P. & Terada, N. Adenine nucleotide translocase 4 is expressed within embryonic ovaries and dispensable during oogenesis. *Reprod. Sci.* **22**, 250–257 (2015)].

· Ref #13 [Polo, J. M. *et al*. Cell type of origin influences the molecular and functional properties of mouse induced pluripotent stem cells. *in Nat. Biotechnol*. 28, 848–855 (2010)] is now [Kokoszka, J. E. *et al*. The ADP/ATP translocator is not essential for the mitochondrial permeability transition pore. *Nature* **427**, 461–465 (2004)].

· Ref #14 [Mangat, J., Lunnon-Wood, T., Rees, P., Elliott, M. & Burch, M. Successful cardiac transplantation in Barth syndrome--single-centre experience of four patients. *Pediatr. Transplant.* **11**, 327–331 (2007)] is now [Perry, R. J. *et al*. Reversal of hypertriglyceridemia, fatty liver disease, and insulin resistance by a liver-targeted mitochondrial uncoupler. *Cell Metab*. **18**, 740–748 (2013)].

· Ref #15 [Huth, S., Jäger, D. & Barth, J. A young fireman candidate with an abnormal chest x-ray]. *Internist (Berl)* **48**, 532–534, 536 (2007)] is now [Tao, H., Zhang, Y., Zeng, X., Shulman, G. I. & Jin, S. Niclosamide ethanolamine-induced mild mitochondrial uncoupling improves diabetic symptoms in mice. *Nat. Med.* **20**, 1263–1269 (2014)].

· Ref #16 [Spencer, C. T. *et al*. Ventricular arrhythmia in the X-linked cardiomyopathy Barth syndrome. *Pediatr. Cardiol.* **26**, 632–637 (2005)] is now [Shabalina, I. G., Kramarova, T. V., Nedergaard, J. & Cannon, B. Carboxyatractyloside effects on brown-fat mitochondria imply that the adenine nucleotide translocator isoforms ANT1 and ANT2 may be responsible for basal and fatty-acid-induced uncoupling respectively. *Biochem. J.* **399**, 405–414 (2006)].

· Ref #17 [Tang, T. *et al*. Combined lifestyle modification and metformin in obese patients with polycystic ovary syndrome. A randomized, placebo-controlled, double-blind multicentre study. *Hum. Reprod.* **21**, 80–89 (2006)] is now [Andreyev A. Yu. *et al*. The ATP/ADP-antiporter is involved in the uncoupling effect of fatty acids on mitochondria. *Eur. J. Biochem.* **182**, 585–592 (1989)].

· Ref #18 [Schlame, M., Ren, M., Xu, Y., Greenberg, M. L. & Haller, I. Molecular symmetry in mitochondrial cardiolipins. *Chem. Phys. Lipids* **138**, 38–49 (2005)] is now [Andreyev A. Yu. *et al*. Carboxyatractylate inhibits the uncoupling effect of free fatty acids. *FEBS Lett*. **226**, 265–269 (1988)].

· Ref #19 [Soyka, M. *et al*. Treatment of alcohol withdrawal syndrome with a combination of tiapride/carbamazepine: results of a pooled analysis in 540 patients. *Eur. Arch. Psychiatry Clin. Neurosci.* **256**, 395–401 (2006)] is now [Lee, Y. S. *et al*. Increased adipocyte O2 consumption triggers HIF–1α, causing inflammation and insulin resistance in obesity. *Cell* **157**, 1339–1352 (2014)].

· Ref #20 [Finsterer, J., Stöllberger, C., Gaismayer, K. & Janssen, B. Acquired noncompaction in Duchenne muscular dystrophy. *Int. J. Cardiol.* **106**, 420–421 (2006)] is now [Chavin, K. D. *et al*. Obesity induces expression of uncoupling protein–2 in hepatocytes and promotes liver ATP depletion. *J. Biol. Chem.* **274**, 5692–5700 (1999)].

· Ref #29 [Huang, S. C. *et al*. Mitral annuloplasty in an infant with Barth syndrome and severe mitral insufficiency: first case report and determination of annular diameter. *J. Thorac. Cardiovasc. Surg.* **136**, 1095–1097 (2008)] is now [Brower, J. V. *et al*. Differential CpG island methylation of murine adenine nucleotide translocase genes. *Biochim. Biophys. Acta* **1789**, 198–203 (2009)].

· Ref #30 [Xu, Y., Sutachan, J. J., Plesken, H., Kelley, R. I. & Schlame, M. Characterization of lymphoblast mitochondria from patients with Barth syndrome. *Lab. Invest.* **85**, 823–830 (2005)] is now [Choi, C. S. *et al*. Continuous fat oxidation in acetyl-CoA carboxylase 2 knockout mice increases total energy expenditure, reduces fat mass, and improves insulin sensitivity. *Proc. Natl Acad. Sci. USA* **104**, 16480–16485 (2007)].

· Ref #31 [Das, R. *et al*. The British Cardiac Society Working Group definition of myocardial infarction: implications for practice. *Heart* **92**, 21–26 (2006)] should be removed.

The correct list of References is as follows:

References

1. Klingenberg, M. The ADP and ATP transport in mitochondria and its carrier. *Biochim. Biophys. Acta* **1778**, 1978–2021 (2008).

2. Kunji, E. R. *et al*. The transport mechanism of the mitochondrial ADP/ATP carrier. *Biochim. Biophys. Acta* **1863**, 2379–2393 (2016).

3. Fiore, C. *et al*. The mitochondrial ADP/ATP carrier: structural, physiological and pathological aspects. *Biochimie* **80**, 137–150 (1998).

4. Monné, M. & Palmieri, F. Antiporters of the mitochondrial carrier family. *Curr. Top. Membr.* **73**, 289–320 (2014).

5. Rodić, N. *et al*. DNA methylation is required for silencing of ant4, an adenine nucleotide translocase selectively expressed in mouse embryonic stem cells and germ cells. *Stem Cells* **23**, 1314–1323 (2005).

6. Graham, B. H. *et al*. A mouse model for mitochondrial myopathy and cardiomyopathy resulting from a deficiency in the heart/muscle isoform of the adenine nucleotide translocator. *Nat. Genet.* **16**, 226–234 (1997).

7. Esposito, L. A., Melov, S., Panov, A., Cottrell, B. A. & Wallace, D. C. Mitochondrial disease in mouse results in increased oxidative stress. *Proc. Natl Acad. Sci. USA* **96**, 4820–4825 (1999).

8. Brower, J. V. *et al*. Evolutionarily conserved mammalian adenine nucleotide translocase 4 is essential for spermatogenesis. *J. Biol. Chem.* **282**, 29658–29666 (2007).

9. Brower, J. V., Lim, C. H., Jorgensen, M., Oh, S. P. & Terada, N. Adenine nucleotide translocase 4 deficiency leads to early meiotic arrest of murine male germ cells. *Reproduction* **138**, 463–470 (2009).

10. Lim, C. H., Brower, J. V., Resnick, J. L., Oh, S. P. & Terada, N. Adenine nucleotide translocase 4 is expressed within embryonic ovaries and dispensable during oogenesis. *Reprod. Sci.* **22**, 250–257 (2015).

11. Levy, S. E., Chen, Y. S., Graham, B. H. & Wallace, D. C. Expression and sequence analysis of the mouse adenine nucleotide translocase 1 and 2 genes. *Gene* **254**, 57–66 (2000).

12. Cho, J. *et al*. Mitochondrial ATP transporter Ant2 depletion impairs erythropoiesis and B lymphopoiesis. *Cell Death Differ.* **22**, 1437–1450 (2015).

13. Kokoszka, J. E. *et al*. The ADP/ATP translocator is not essential for the mitochondrial permeability transition pore. *Nature* **427**, 461–465 (2004).

14. Perry, R. J. *et al*. Reversal of hypertriglyceridemia, fatty liver disease, and insulin resistance by a liver-targeted mitochondrial uncoupler. *Cell Metab.* **18**, 740–748 (2013).

15. Tao, H., Zhang, Y., Zeng, X., Shulman, G. I. & Jin, S. Niclosamide ethanolamine-induced mild mitochondrial uncoupling improves diabetic symptoms in mice. *Nat. Med.* **20**, 1263–1269 (2014).

16. Shabalina, I. G., Kramarova, T. V., Nedergaard, J. & Cannon, B. Carboxyatractyloside effects on brown-fat mitochondria imply that the adenine nucleotide translocator isoforms ANT1 and ANT2 may be responsible for basal and fatty-acid-induced uncoupling respectively. *Biochem. J.* **399**, 405–414 (2006).

17. Andreyev A. Yu. *et al*. The ATP/ADP-antiporter is involved in the uncoupling effect of fatty acids on mitochondria. *Eur. J. Biochem.* **182**, 585–592 (1989).

18. Andreyev A. Yu. *et al*. Carboxyatractylate inhibits the uncoupling effect of free fatty acids. *FEBS Lett.* **226**, 265–269 (1988).

19. Lee, Y. S. *et al*. Increased adipocyte O_2_ consumption triggers HIF-1α, causing inflammation and insulin resistance in obesity. *Cell* **157**, 1339–1352 (2014).

20. Chavin, K. D. *et al*. Obesity induces expression of uncoupling protein-2 in hepatocytes and promotes liver ATP depletion. *J. Biol. Chem.* **274**, 5692–5700 (1999).

21. Ko, Y. H., Delannoy, M., Hullihen, J., Chiu, W. & Pedersen, P. L. Mitochondrial ATP synthasome. Cristae-enriched membranes and a multiwell detergent screening assay yield dispersed single complexes containing the ATP synthase and carriers for Pi and ADP/ATP. *J. Biol. Chem.* **278**, 12305–12309 (2003).

22. Grimm, D. *et al*. Preclinical in vivo evaluation of pseudotyped adeno-associated virus vectors for liver gene therapy. *Blood* **102**, 2412–2419 (2003).

23. High, K. A. & Anguela, X. M. Adeno-associated viral vectors for the treatment of hemophilia. *Hum. Mol. Genet.* **25**, R36–R41 (2016).

24. Wang, L. *et al*. Comparative Study of Liver Gene Transfer With AAV Vectors Based on Natural and Engineered AAV Capsids. *Mol. Ther.* **23**, 1877–1887 (2015).

25. Schiebel, K., Weiss, B., Wöhrle, D. & Rappold, G. A human pseudoautosomal gene, ADP/ATP translocase, escapes X-inactivation whereas a homologue on Xq is subject to X-inactivation. *Nat. Genet.* **3**, 82–87 (1993).

26. Chevrollier, A., Loiseau, D., Reynier, P. & Stepien, G. Adenine nucleotide translocase 2 is a key mitochondrial protein in cancer metabolism. *Biochim. Biophys. Acta* **1807**, 562–567 (2011).

27. Rinella, M. E. Nonalcoholic fatty liver disease: a systematic review. *JAMA* **313**, 2263–2273 (2015).

28. Misra, V. L., Khashab, M. & Chalasani, N. Nonalcoholic fatty liver disease and cardiovascular risk. *Curr. Gastroenterol. Rep.* **11**, 50–55 (2009).

29. Brower, J. V. *et al*. Differential CpG island methylation of murine adenine nucleotide translocase genes. *Biochim. Biophys. Acta* **1789**, 198–203 (2009).

30. Choi, C. S. *et al*. Continuous fat oxidation in acetyl-CoA carboxylase 2 knockout mice increases total energy expenditure, reduces fat mass, and improves insulin sensitivity. *Proc. Natl Acad. Sci. USA* **104**, 16480–16485 (2007).

