# Supplementary material for: Mitochondrial ATP transporter depletion protects mice against liver steatosis and insulin resistance
Source: Nat Commun. 2017 Feb 16;8:14477. doi: 10.1038/ncomms14477 (PMC5316896; doi:10.1038/ncomms14477)
Supplement: Supplementary Information — Supplementary figures and supplementary table. [file ncomms14477-s1.pdf]

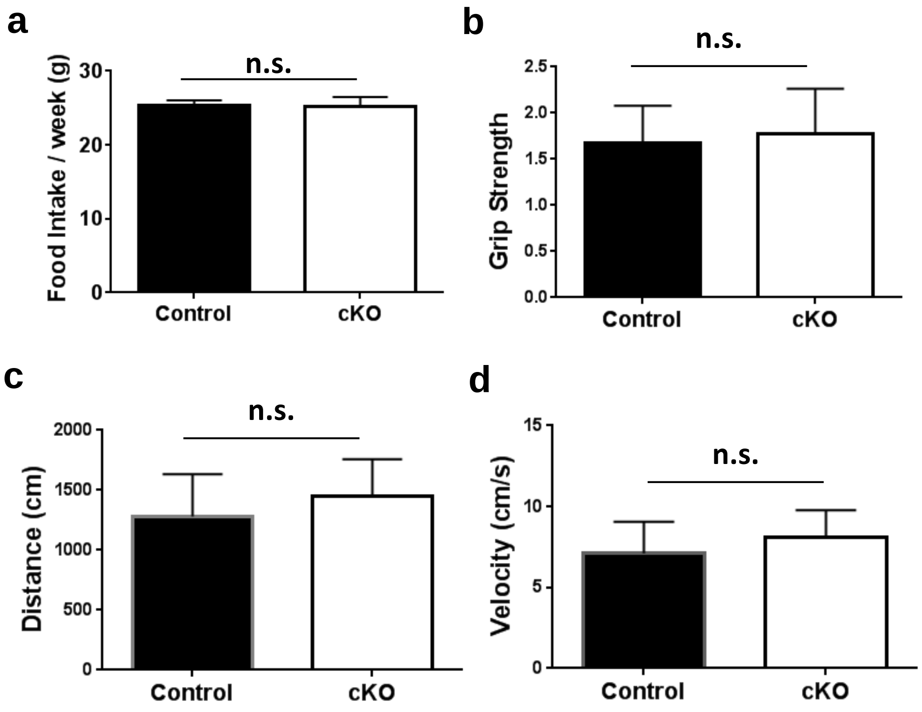

Supplementary Figure 1

Supplementary figure 1: Food intake and physical capacities. (**a**) Amount of food intake weekly (n=20, t-test, error bars=s.d.). (**b**) Grip strength (n=6, t-test, error bars=s.d.). (**c**) Total running distance (n=6, t-test, error bars=s.d.). (**d**) Running velocity (n=6, t-test, error bars=s.d.).

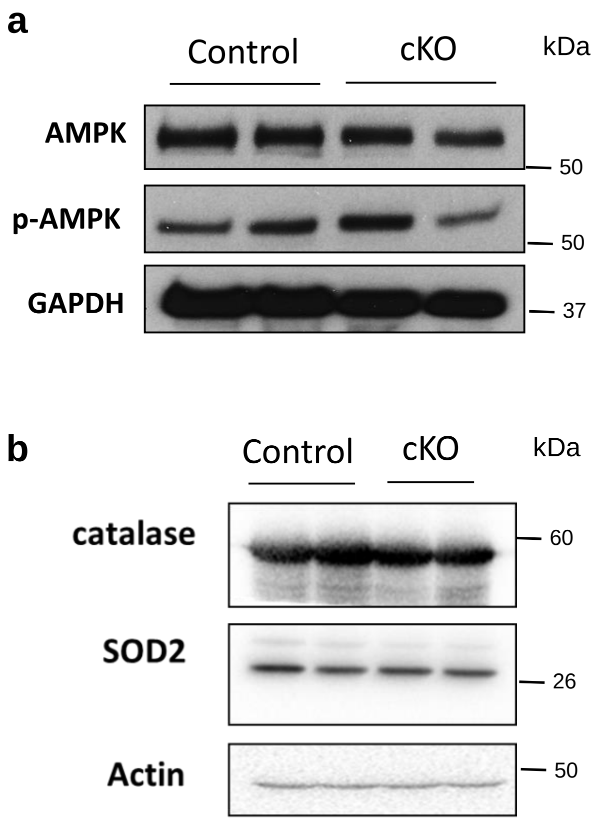

Supplementary Figure 2

Supplementary figure 2: Cellular AMPK and antioxidant enzymes. (**a**) Immunoblot analysis for total AMPK, phosphorylated AMPK and GAPDH expression in liver lysates. (**b**) Immunoblot analysis for catalase, superoxide dismutase 2 (SOD2), and β-actin expression in liver lysates.

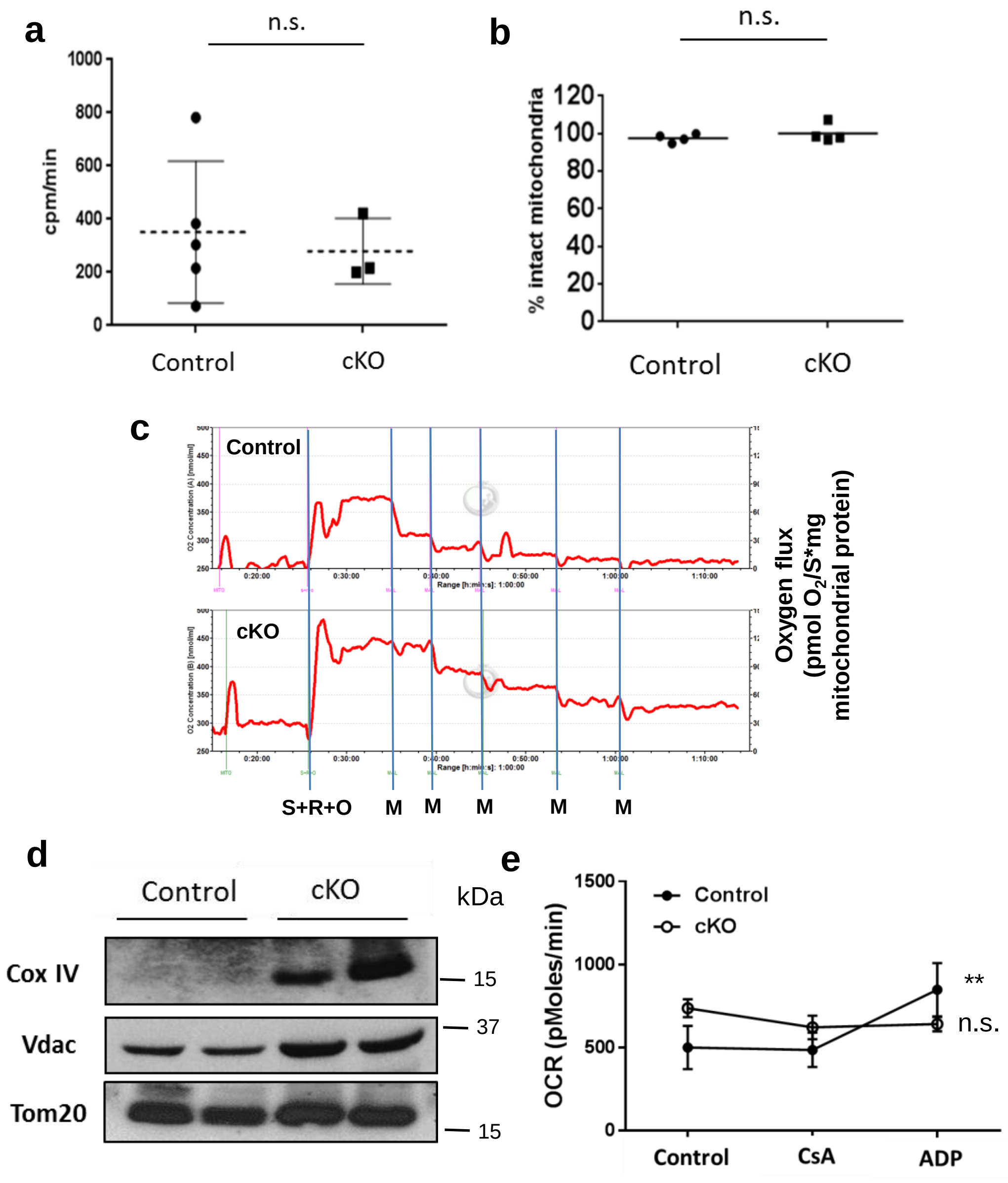

Supplementary Figure 3

Supplementary figure 3: Inner/outer membrane integrity and respiration measurement in *Ant2*-deleted liver mitochondria. (**a**) Pyruvate uptake capacity measured by [^{14}C]Pyruvate uptake in isolated liver mitochondria (n=3~5, t-test, error bars=s.d.). (**b**) Oxidation of reduced cytochrome c in isolated liver mitochondria (n=4, t-test, error bars=s.d.). (**c**) Representative traces of oxygen flux of liver mitochondria assessed with the Oroboros O2k respirometer in the presence of succinate, rotenone, and oligomycin (S+R+O), and malonate (M) (n=4). (**d**) Immunoblot analysis in isolated mitochondria for cytochrome c oxidase (Cox) IV, voltage dependent anion channel (Vdac), and Tom20. (e) Mitochondrial OCR assessed with XF24 extracellular flux analyzer (Seahorse) under sequential treatment of CsA and ADP (n=4; **$P<0.01$ by one-way ANOVA, error bars=s.d.).

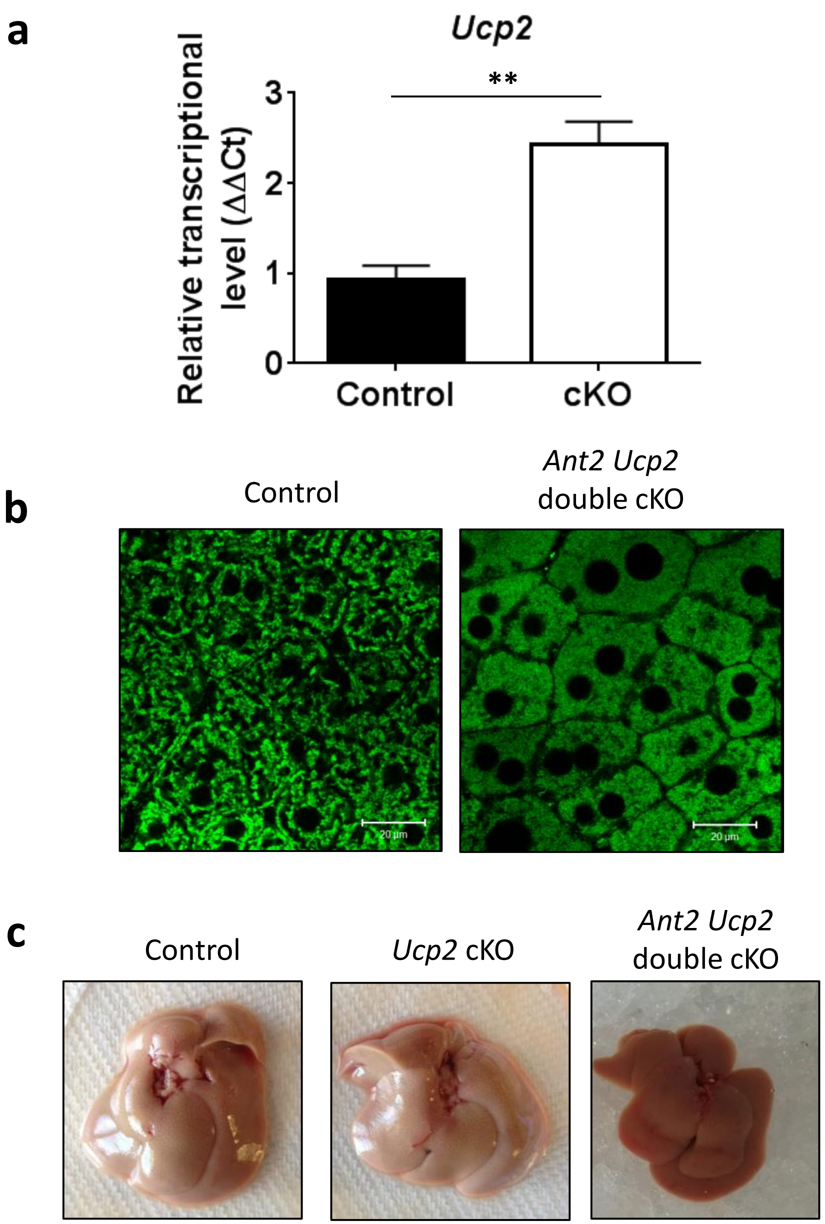

Supplementary Figure 4

Supplementary figure 4: Phenotypes of *Ucp2* cKO liver and mitochondria. (**a**) qRT-PCR analysis for *Ucp2* mRNA expression in the liver (n=3; **$P<0.01$ by t-test, error bars=s.d.). (**b**) Multiphoton microscopy analysis after the liver was perfused with Rhodamine-123 (n=3). Scale bar; 20 µm. (**c**) Liver gross appearance under a high fat/high fructose diet (8 weeks) (n>4).

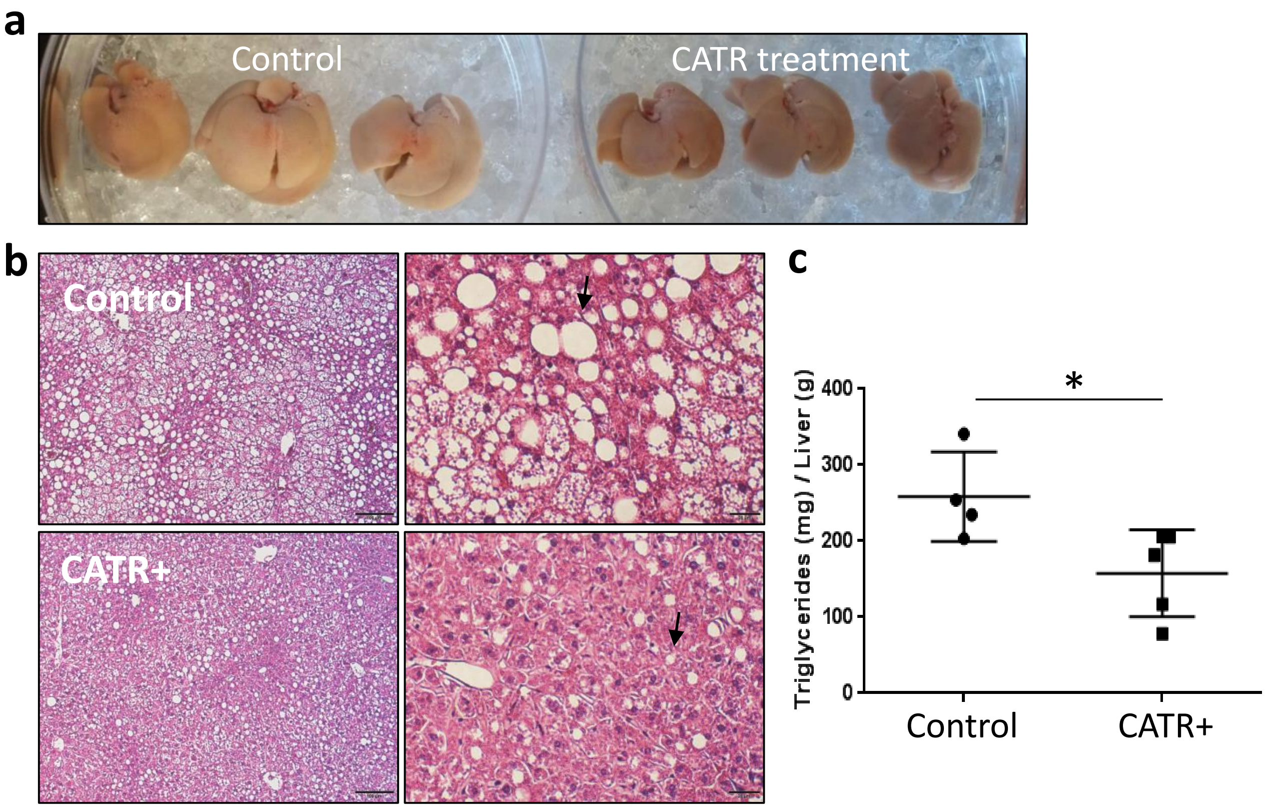

Supplementary Figure 5

Supplementary figure 5: Systemic CATR treatment a high fat and high fructose diet. Wild type C57BL6 mice were subjected to a high fat (40%)/high fructose (20%) diet for 8 weeks. During the last two weeks, CATR (1 mg/kg) or vehicle (PBS) (Control) was administered daily by intraperitoneal injection. (**a**) Liver gross appearance. (**b**) Liver histology analysis with H&E staining. Scale bars; 100 μm (left panels) and 20 μm (right panels). (**c**) Total triglyceride levels in the liver (*P<0.05 by t-test, error bars=s.d.).

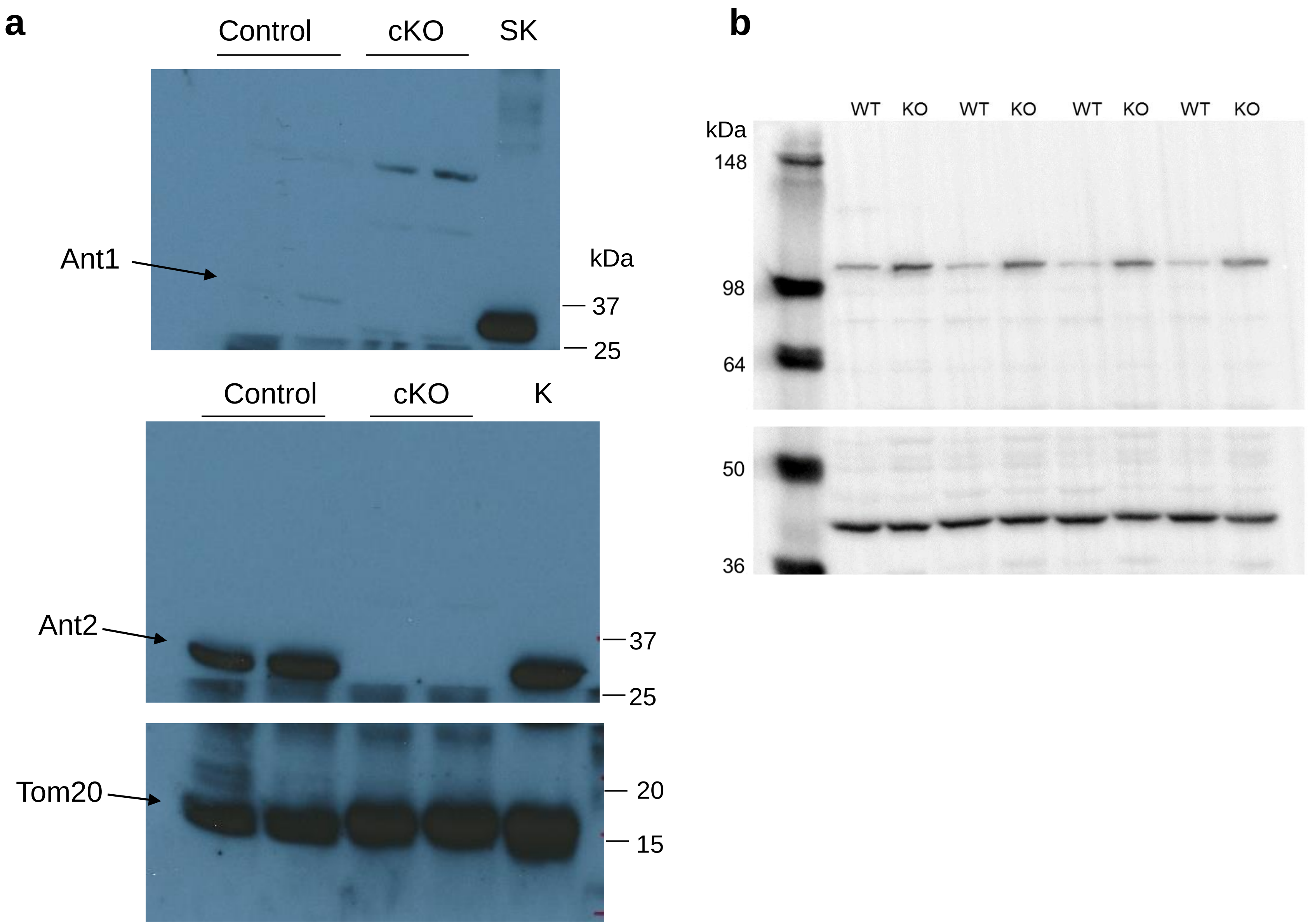

Supplementary Figure 6

Supplementary figure 6: Uncropped scan of critical immunoblots. Original uncropped scan data of Fig. 1b (**a**) and Fig. 2d (**b**) are shown here.

**Blood Chemistry**	**Control**	***Ant2*cKO**
ALT (U/L)	58.8 ± 16.4	69.1 ± 29.9
AST (U/L)	235.8 ± 124.1	241.9 ± 102.4
Albumin (g/dL)	3.9 ± 0.3	3.8 ± 0.3
Bilirubin (mg/dL)	0.5 ± 0.2	0.6 ± 0.2
Total Protein (g/dL)	6.2 ± 0.3	5.7 ± 0.5
Lactate (mM)	6.3 ± 1.7	6.7 ± 2
Glucose** (mg/dL)	212.6 ± 103.7	125.2 ± 9
Cholesterol** (mg/dL)	130.0 ± 29.1	81.8 ± 16.2
Insulin** (ng/ml)	1071 ± 251	470 ± 99
Ketone Body*** (µM)	111.2 ± 49.4	325.2 ± 128.0
Urea* (mM)	6.1 ± 1.2	5.0 ± 1.2

Supplementary Table 1: Blood chemistry of control and Ant2 cKO mice (16-18 weeks old, n>10) *P < 0.05, **p<0.01, ***p<0.001
